# Supplementary material for: An additive Gaussian process regression model for interpretable non-parametric analysis of longitudinal data
Source: Nat Commun. 2019 Apr 17;10:1798. doi: 10.1038/s41467-019-09785-8 (PMC6470127; doi:10.1038/s41467-019-09785-8)
Supplement: Supplementary file 1 — Supplementary Information [file 41467_2019_9785_MOESM1_ESM.pdf]

Supplementary Information

An additive Gaussian process regression model for interpretable non-parametric analysis of longitudinal data

Lu Cheng et al.

March 19, 2019

Contents

Supplementary Figures **2**

Supplementary Tables **8**

Supplementary Methods **10**

Supplementary Method 1: Methods for comparison in the simulation experiments 10

Linear mixed-effects model (LME) 10

Linear mixed-effects model (LME) with polynomial terms 12

Gaussian process with automatic relevance determination (ARD) kernel 14

Supplementary Method 2: LonGP algorithm 15

Supplementary Method 3: MCMC details 17

Supplementary Method 4: Software architecture 17

Supplementary Notes **18**

Supplementary Note 1: Full comparison results on simulated datasets 18

Linear mixed-effects (LME) model 18

Linear mixed-effects model (LME) with polynomial terms 21

Gaussian process with automatic relevance determination (ARD) kernel 23

Supplementary Figures

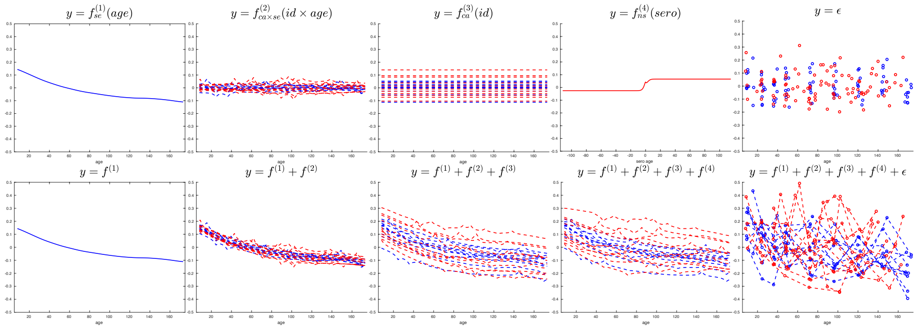

Supplementary Figure 1: Predicted components and cumulative effects for protein P07602 (Proteomics dataset). Top panel shows contributions of individual components and lower panel shows cumulative effects. Red lines correspond to cases and blue lines correspond to controls. Bottom right panel shows the (centred) data. Note, the x-axis of $f^{(4)}$ is time relative to seroconversion age.

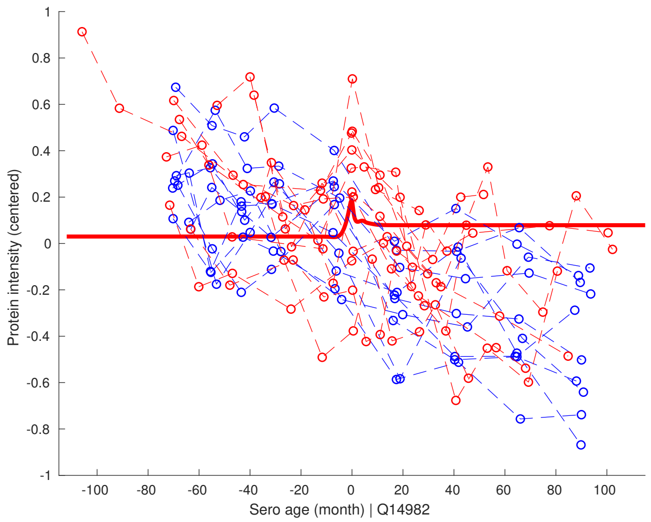

Supplementary Figure 2: Predicted mean of the *sero* component for protein Q14982 (Proteomics dataset). The dashed red lines show the measurements of cases and the dashed blue lines are measurements of controls. x-axis is time relative to seroconversion age and y-axis is centred protein intensity. Mean seroconversion age of all cases (79.42 month) is used as the seroconversion age for controls. The solid red line corresponds to the mean of the seroconversion component $y = f_{ns}^{(4)}(sero)$.

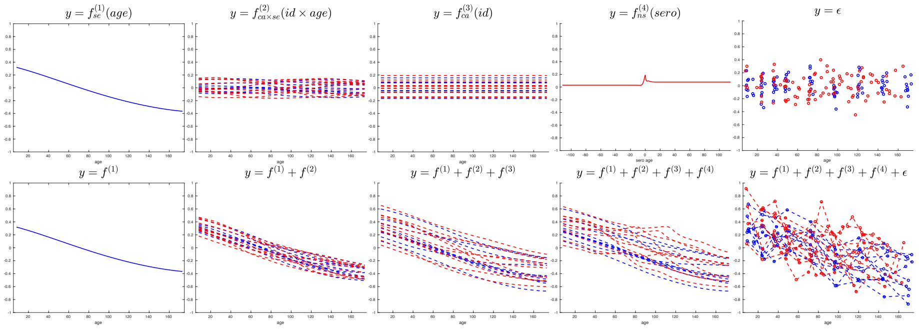

Supplementary Figure 3: Predicted components and cumulative effects for protein Q14982 (Proteomics dataset). Top panel shows contributions of individual components and lower panel shows cumulative effects. Red lines correspond to cases and blue lines correspond to controls. Bottom right panel shows the (centred) data. Note the x-axis of $f^{(4)}$ is time relative to seroconversion age.

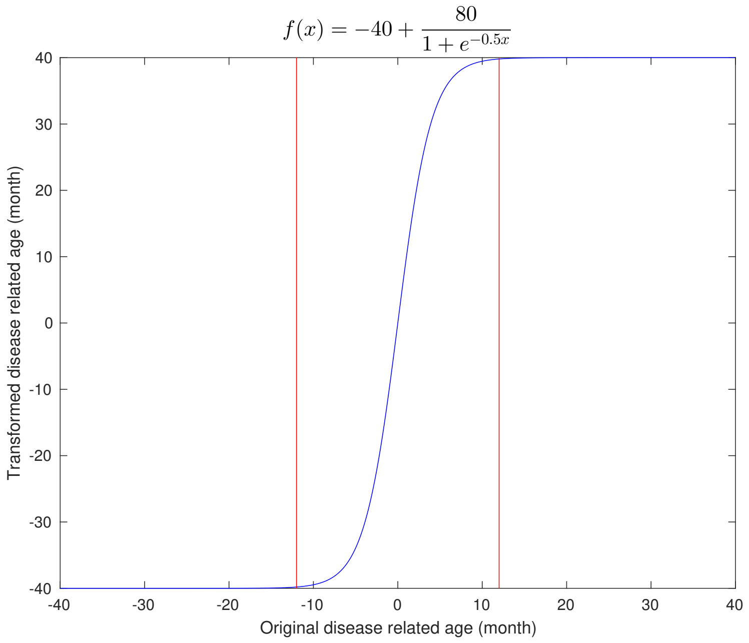

Supplementary Figure 4: Non-stationary transformation. The x-axis is the original disease related age and the y-axis is the transformed disease related age. Sigmoid function $f(x) = -40 + \frac{80}{1+e^{-0.5x}}$ is used for the transformation. The red bars indicate the positions of ± 12 month.

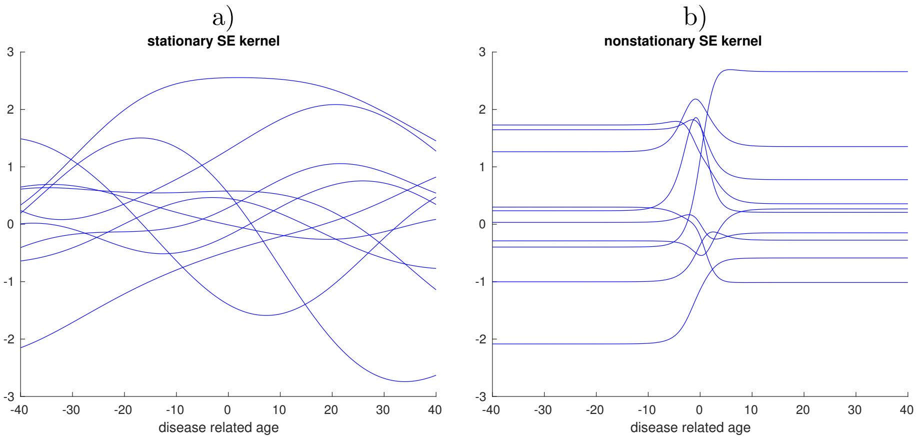

Supplementary Figure 5: Functions drawn from stationary and non-stationary SE (square exponential) kernel. a) Functions drawn from a stationary SE kernel with length-scale $l_{\text{se}} = 1$ and magnitude $\sigma_{se}^2 = 1$. b) Functions drawn form a non-stationary SE kernel by first applying the transformation shown in Supplementary Figure 4 and then generated using the same SE kernel with scale $l_{\text{se}} = 1$ and magnitude $\sigma_{se}^2 = 1$. Random functions are drawn using the standardised inputs and then transformed back to original range.

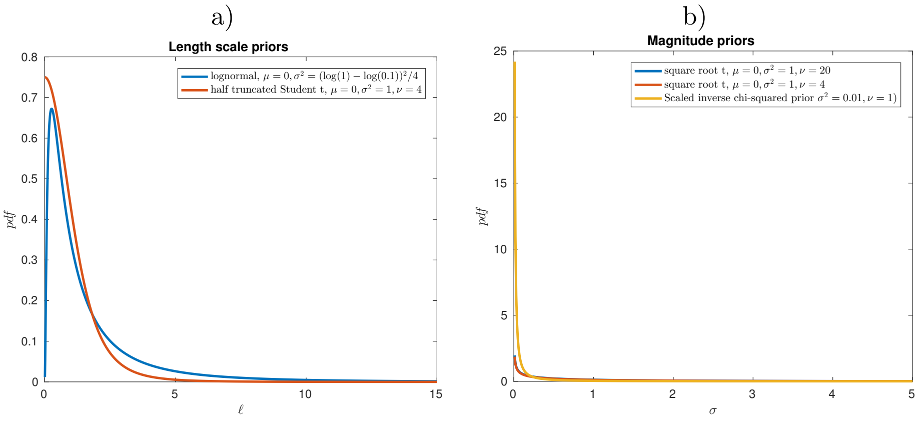

Supplementary Figure 6: Priors for kernel parameters. a) Priors for length-scales and b) priors for magnitude and noise variance. Note, that the target variable and continuous covariates are all standardised to mean 0 and standard deviation 1.

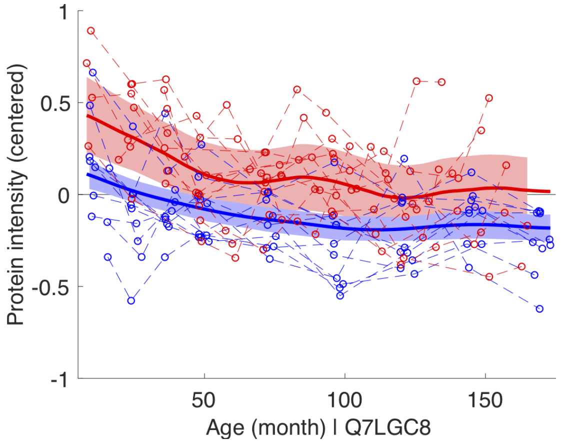

Supplementary Figure 7: Cumulative effect $y = f_{\text{se}}^{(1)}(age) + f_{\text{bi}}^{(2)}(group) + f_{\text{bi}\times\text{se}}^{(3)}(group \times age)$ against real (centred) intensity of protein Q7LGC8 (Proteomics dataset). Red lines are cases and blue lines are controls. The red and blue shaded areas are the $\pm\sigma$ of the predictive distribution on the original training data for the cases and controls, where σ refers to the pointwise standard deviation of the predictive distribution.

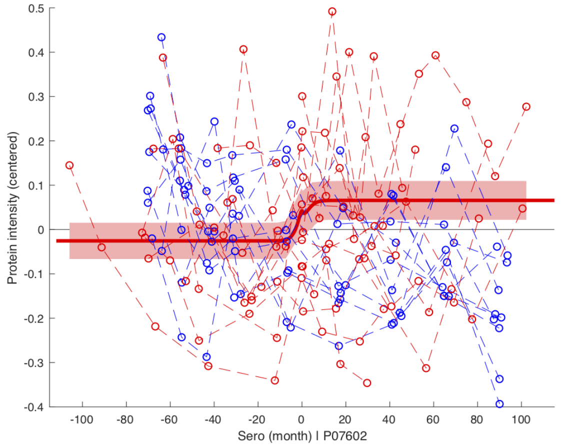

Supplementary Figure 8: Predicted mean of the *sero* component for protein P07602 (Proteomics dataset). The dashed red lines show the measurements of cases and the dashed blue lines are controls. x-axis indicates time from seroconversion and y-axis is the centred protein intensity. Mean seroconversion age of all cases (79.42 month) is used as the seroconversion age for controls. The solid red line corresponds to the mean of the seroconversion component $y = f_{\text{ns}}^{(4)}(sero)$. The red and blue shadow areas are the $\pm\sigma$ of the predictive distribution on the original training data for the cases and controls, where σ refers to the pointwise standard deviation of the predictive distribution.

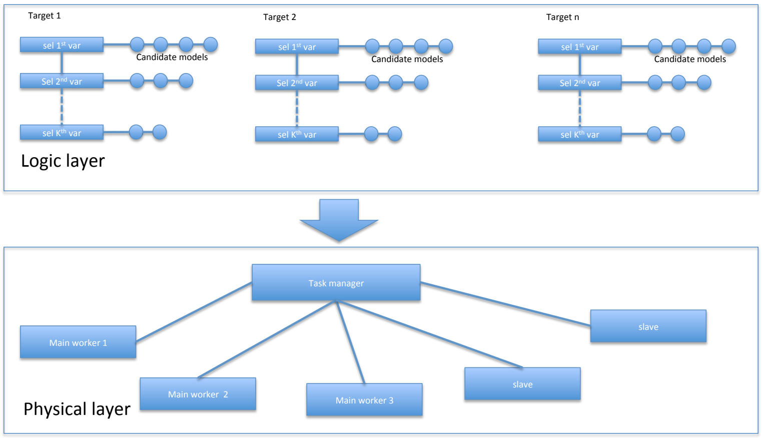

Supplementary Figure 9: Software architecture. The task manager monitors the whole process and schedules the tasks. The main worker ensures the tasks for a given target is executed in the right order. The slaves run parallel jobs assigned by the task managers.

Supplementary Tables

Supplementary Table 1. Inclusion of *diseaseAge* in the final model as a function of noise variance. Table shows the number of times the *diseaseAge* covariate is included in the inferred model among 100 Monte Carlo simulations (Simulated dataset).

Generated Datasets	noise = 1	noise = 3	noise = 5	noise = 8
AGPM1	0	0	5	0
AGPM2	0	1	0	2
AGPM3	0	0	1	2
AGPM4	98	97	98	97
AGPM5	99	97	94	92

Supplementary Table 2. Inclusion of *diseaseAge* in the final model as a function of sample size. Table shows the number of times the *diseaseAge* covariate is included in the inferred model among 100 Monte Carlo simulations (Simulated dataset).

Generated Datasets	10 cases and 10 controls	20 cases and 20 controls	30 cases and 30 controls	40 cases and 40 controls
AGPM1	4	0	0	0
AGPM2	0	1	0	5
AGPM3	0	0	0	0
AGPM4	94	97	99	96
AGPM5	93	97	100	100

Supplementary Table 3. Model selection accuracy as a function of sampling time points. Table shows the number of times the correct model is identified among 100 Monte Carlo simulations. (Simulated dataset)

Generated Datasets	2 months	3 months	4 months	6 months
AGPM1	97	98	94	96
AGPM2	95	95	88	85
AGPM3	97	95	91	93
AGPM4	96	92	86	86
AGPM5	94	88	87	86

Supplementary Table 4. Inclusion of *diseaseAge* in the final model as a function of sampling time points. Table shows the number of times the *diseaseAge* covariate is included in the inferred model among 100 Monte Carlo simulations (Simulated dataset).

Generated Datasets	2 months	3 months	4 months	6 months
AGPM1	0	0	0	0
AGPM2	0	1	3	4
AGPM3	0	0	1	1
AGPM4	100	97	94	92
AGPM5	98	97	94	92

Supplementary Methods

Supplementary Method 1: Methods for comparison in the simulation experiments

This section provides a description of the implementation of the comparison methods in the simulation experiments. The input covariates are *age*, *diseaseAge*, *group*, *loc*, *gen*, and *id*, where *age* and *diseaseAge* are continuous and the rest are either binary or categorical. The output/target variable is denoted by y here.

Linear mixed-effects model (LME)

We first used the following linear mixed-effect model [1]:

$$y = \beta_0 + \sum_{i=1}^{n_f} \beta_i x_i + \sum_{i=1}^{n_r} b_i z_i + \epsilon, \tag{1}$$

where x_i is one of the n_f covariates with fixed effects, z_i is one of the n_r covariates with random effects, β_i and b_i are the corresponding linear coefficients and ϵ is the *i.i.d* Gaussian noise. The key idea of LME is that β_i are independent of each other and is shared by all individuals, while b_i can be correlated with each other and assumed to be drawn from a multivariate Gaussian.

$$\boldsymbol{b} = (b_1, b_2, \ldots, b_{n_r})^T \sim N(\mathbf{0}, \sigma^2 D(\theta)), \tag{2}$$

where σ^2 controls the variance and $D(\theta)$ parameterises the full n_r-by-n_r correlation matrix. Maximum likelihood estimates are then obtained for all parameters in Eq. (1).

Here we use the LME model to incorporate the same covariates and their interactions as in `lonGP`. We model shared age and disease age effects, location, gender, and disease group effects as fixed effects. These are shown as single linear terms such as *age*, *dise*, *group*, *loc*, and *gen*. Note, that we have used *dise* to replace *diseAge* for brevity. The age effects specific to location, gender and disease group are also modelled as fixed effects which are shown as interaction terms, such as $loc \times age$, $gender \times age$, $group \times age$. The individual differences are modelled as random effects, shown as $id \times age$ and id. Since there are 5 free

covariates, there are $2^5 = 32$ models in total. We list all the models as follows:

$$y \sim 1 + [id]$$
$$y \sim 1 + age + [age \times id + id]$$
$$y \sim 1 + dise + [id]$$
$$y \sim 1 + age + dise + [age \times id + id]$$
$$y \sim 1 + loc + [id]$$
$$y \sim 1 + age + loc + loc \times age + [age \times id + id]$$
$$y \sim 1 + dise + loc + [id]$$
$$y \sim 1 + age + dise + loc + loc \times age + [age \times id + id]$$
$$y \sim 1 + gen + [id]$$
$$y \sim 1 + age + gen + gen \times age + [age \times id + id]$$
$$y \sim 1 + dise + gen + [id]$$
$$y \sim 1 + age + dise + gen + gen \times age + [age \times id + id]$$
$$y \sim 1 + loc + gen + [id]$$
$$y \sim 1 + age + loc + gen + gen \times age + loc \times age + [age \times id + id]$$
$$y \sim 1 + dise + loc + gen + [id]$$
$$y \sim 1 + age + dise + loc + gen + gen \times age + loc \times age + [age \times id + id]$$
$$y \sim 1 + group + [id]$$
$$y \sim 1 + age + group + group \times age + [age \times id + id]$$
$$y \sim 1 + dise + group + [id]$$
$$y \sim 1 + age + dise + group + group \times age + [age \times id + id]$$
$$y \sim 1 + loc + group + [id]$$
$$y \sim 1 + age + loc + group + group \times age + loc \times age + [age \times id + id]$$
$$y \sim 1 + dise + loc + group + [id]$$
$$y \sim 1 + age + dise + loc + group + group \times age + loc \times age + [age \times id + id]$$
$$y \sim 1 + gen + group + [id]$$
$$y \sim 1 + age + gen + group + group \times age + gen \times age + [age \times id + id]$$
$$y \sim 1 + dise + gen + group + [id]$$
$$y \sim 1 + age + dise + gen + group + group \times age + gen \times age + [age \times id + id]$$
$$y \sim 1 + loc + gen + group + [id]$$
$$y \sim 1 + age + loc + gen + group + group \times age + gen \times age + loc \times age + [age \times id + id]$$
$$y \sim 1 + dise + loc + gen + group + [id]$$
$$y \sim 1 + age + dise + loc + gen + group + group \times age + gen \times age + loc \times age + [age \times id + id],$$

where the terms in the brackets are random effects and other terms are fixed effects. Linear coefficients are omitted for clarity.

For model selection, we use 10-fold cross validation to fit each model and make predictions, then calculate the root-mean-square error (RMSE) for each

model. After that, we compare the RMSE of each model and choose the one with the lowest RMSE.

We use the MATLAB (version 2017b) function `fitlme` for the implementation. The code is available at `https://github.com/chengl7/LonGP/blob/master/comparison/lmm/lmm_main.m`.

Linear mixed-effects model (LME) with polynomial terms

In order to bring non-linearity into the modelling, we try to include a second order term for the continuous covariates in our models, i.e., age^2 and $dise^2$. The 32 models used are given as follows:

$$
\begin{aligned}
y &\sim 1+[id]\\
y &\sim 1+age^2+age+[age\times id+id]\\
y &\sim 1+dise^2+dise+[id]\\
y &\sim 1+age^2+age+dise^2+dise+[age\times id+id]\\
y &\sim 1+loc+[id]\\
y &\sim 1+age^2+age+loc+loc\times age+[age\times id+id]\\
y &\sim 1+dise^2+dise+loc+[id]\\
y &\sim 1+age^2+age+dise^2+dise+loc+loc\times age+[age\times id+id]\\
y &\sim 1+gen+[id]\\
y &\sim 1+age^2+age+gen+gen\times age+[age\times id+id]\\
y &\sim 1+dise^2+dise+gen+[id]\\
y &\sim 1+age^2+age+dise^2+dise+gen+gen\times age+[age\times id+id]\\
y &\sim 1+loc+gen+[id]\\
y &\sim 1+age^2+age+loc+gen+gen\times age+loc\times age+[age\times id+id]\\
y &\sim 1+dise^2+dise+loc+gen+[id]\\
y &\sim 1+age^2+age+dise^2+dise+loc+gen+gen\times age+loc\times age+[age\times id+id]\\
y &\sim 1+group+[id]\\
y &\sim 1+age^2+age+group+group\times age+[age\times id+id]\\
y &\sim 1+dise^2+dise+group+[id]\\
y &\sim 1+age^2+age+dise^2+dise+group+group\times age+[age\times id+id]\\
y &\sim 1+loc+group+[id]\\
y &\sim 1+age^2+age+loc+group+group\times age+loc\times age+[age\times id+id]\\
y &\sim 1+dise^2+dise+loc+group+[id]\\
y &\sim 1+age^2+age+dise^2+dise+loc+group+group\times age+loc\times age+[age\times id+id]\\
y &\sim 1+gen+group+[id]\\
y &\sim 1+age^2+age+gen+group+group\times age+gen\times age+[age\times id+id]\\
y &\sim 1+dise^2+dise+gen+group+[id]\\
y &\sim 1+age^2+age+dise^2+dise+gen+group+group\times age+gen\times age+[age\times id+id]\\
y &\sim 1+loc+gen+group+[id]\\
y &\sim 1+age^2+age+loc+gen+group+group\times age+gen\times age+loc\times age\\
&\quad +[age\times id+id]\\
y &\sim 1+dise^2+dise+loc+gen+group+[id]\\
y &\sim 1+age^2+age+dise^2+dise+loc+gen+group+group\times age\\
&\quad +gen\times age+loc\times age+[age\times id+id],
\end{aligned}
$$

where the terms in the brackets are random effects and other terms are fixed effects. Linear coefficients are omitted for clarity. The same procedures as described in the previous section are applied for selecting the best model. The implementation can be found at `https://github.com/chengl7/LonGP/blob/master/comparison/lmm/lmm_main1.m`.

Gaussian process with automatic relevance determination (ARD) kernel

Gaussian process with automatic relevance determination (ARD) kernel [2] is a standard method for feature selection with GPs. The ARD kernel for two inputs $\boldsymbol{x} = (x_1, x_2, \ldots, x_D)$ and $\boldsymbol{x'} = (x'_1, x'_2, \ldots, x'_D)$ is given as follows:

$$k(\boldsymbol{x}, \boldsymbol{x'}) = \exp\left(-\frac{1}{2}\sum_{j=1}^{D}\frac{(x_j - x'_j)^2}{l_j^2}\right), \tag{3}$$

where l_j is the length scale for the jth covariate and D is the total number of covariates. ARD was originally proposed for continuous covariates but since longitudinal biomedical studies often involve discrete covariates as well, we modify and apply ARD here for a combination of continuous and discrete covariates as follows. We first normalise the continuous covariates, *age* and *diseaseAge* to mean $\mu = 0$ and standard deviation $\sigma = 1$. For discrete covariates, we define the squared Euclidean distance as follows: if $x_j = x'_j$, then we use $(x_j - x'_j)^2 = 0$, and if $x_j \neq x'_j$ then we define $(x_j - x'_j)^2 = d$. We empirically set d to a value of 4. The idea is to assign maximal correlation to discrete data items with the same value and an appropriate smaller correlation to data items with different values, such that all covariates have similar variances and length scales become comparable for feature selection.

The importance of a covariate lies on its linear coefficient $\lambda_j = \frac{1}{l_j^2}$ in the ARD kernel. The larger the length scale the smaller the linear coefficient, and thus is an indication of a less important covariate. We normalise the linear coefficients by

$$\tilde{\lambda}_k = \frac{\lambda_k}{\sum_{j=1}^{D}\lambda_j}, \tag{4}$$

then select the covariate if $\tilde{\lambda}_k$ is greater than 0.05. The covariate *id* is always included for ease of comparison with other methods.

We use MATLAB (version 2017b) function `fitrgp` for the implementation, where we have implemented a customised kernel function for the ARD kernel as described above. The type-II maximum likelihood estimates are then obtained by numerical optimisation using the `quasinewton` optimiser. The code is available at `https://github.com/chengl7/LonGP/blob/master/comparison/ard/ard_main.m`.

Supplementary Method 2: LonGP algorithm

This section describes in detail how the covariate selection process works. Let us denote a given set of continuous covariates by $\mathbf{C} = (V_1, V_2, ..., V_c)$ and the discrete covariates by $\mathbf{B} = (V_{c+1}, V_{c+2}, ..., V_{c+b})$, where c and b are the number of continuous and binary/categorical variables. The categorical covariate *id* must be included in set **B**. In `LonGP`, the user needs to provide the kernel types (Sec. 2.4) for all the given covariates, as well as indicate whether interactions for each covariate are allowed. The data is automatically standardised and the parameter priors for kernels are predefined (see Sec. 2.5). For any given subset of covariates (must include *id*), the additive GP model is constructed by the following rules:

1. Construct a kernel for each covariate according to the given kernel type and add it to the model.
2. For each continuous covariate that allows interaction, construct product kernels with all categorical/binary covariates that also allow interactions (and that are also covariates of a given model) and add them to the model.
3. For each pair of categorical/binary covariates (excluding *id*) that allows interactions, construct a product kernel and add it to the model.
4. Add the noise to finalise the model.

For any covariate subset **V**, we can construct a GP model GPM(**V**) according

to these four steps. The covariates are then selected by the following algorithm:

Algorithm 1: Stepwise GP regression algorithm

Result: A GP model
Set the current selected covariate set to $\mathbf{V}_{\text{curr}} = \{id\}$ and the current model to GPM($\mathbf{V}_{\text{curr}}$), infer the parameters using MCMC and perform LOOCV ;
for $i \leftarrow 1$ **to** c **do**
    foreach $V_j \in \mathbf{C} \setminus \mathbf{V}_{curr}$ **do**
        Add V_j and build a candidate model GPM($\mathbf{V}_{\text{curr}} \cup V_j$), run MCMC and perform LOOCV ;
    end
    Compare all the generated candidate models (Section 2.7.3) and choose the best model GPM($\mathbf{V}_{\text{curr}} \cup V_{\text{best}}$) ;
    Calculate LOOCVF of GPM($\mathbf{V}_{\text{curr}} \cup V_{\text{best}}$) versus GPM($\mathbf{V}_{\text{curr}}$) ;
    if $LOOCVF \geq 0.8$ **then**
        Set $\mathbf{V}_{\text{curr}} = \mathbf{V}_{\text{curr}} \cup V_{\text{best}}$, update the current model accordingly ;
    else
        break ;
    end
end
Perform SCV on the current model ;
for $i \leftarrow 1$ **to** b **do**
    foreach $V_j \in \mathbf{B} \setminus \mathbf{V}_{curr}$ **do**
        Add V_j and build a candidate model GPM($\mathbf{V}_{\text{curr}} \cup V_j$), use numerical integration with CCD and perform SCV ;
    end
    Compare all the generated candidate models (Section 2.7.3) and choose the best model GPM($\mathbf{V}_{\text{curr}} \cup V_{\text{best}}$) ;
    Calculate SCVF of GPM($\mathbf{V}_{\text{curr}} \cup V_{\text{best}}$) versus GPM($\mathbf{V}_{\text{curr}}$) ;
    if $SCVF \geq 0.95$ **then**
        Set $\mathbf{V}_{\text{curr}} = \mathbf{V}_{\text{curr}} \cup V_{\text{best}}$, update the current model accordingly ;
    else
        break ;
    end
end
Make the current model the final model and run MCMC inference. ;
Make predictions using each component (kernel) on the training data, calculate the variances. ;
Calculate the explained variance (variances divided by the sum) of each component, delete components that have lower variances than a user defined threshold ;

The algorithm tries to select covariates with reasonably large effects and the thresholds of the LOOCVF and SCVF are determined by the user (defaults are 0.8 and 0.95).

Supplementary Method 3: MCMC details

We start 4 independent Markov chains from different, randomly initialised initial parameter values. Then, we combine the 4 chains and check the convergence by throwing away 500 burn-in samples and thinning the remaining 2000 samples by 5. If converged, then quit; otherwise we thin the combined chain further by 2. If not converged, we repeat the process and check the convergence from the resulting combined markov chains, for at most 4 times. The potential reduction scaling factor (PRSF) [3] R is used to check the convergence by the following rules: if $R <= 1.1$, converged; if $1.1 < R <= 1.2$, does not converge well; if $R > 1.2$, does not converge.

Supplementary Method 4: Software architecture

In many occasions more than one target variable is measured, such as in transcriptome studies using microarrays or RNA-sequencing, which means that we need to run `LonGP` for many target variables at the same time. Fortunately, several parts of our method can be efficiently parallelised. We designed the `LonGP` software package so that it can be easily deployed and parallelised in a modern computing cluster with shared storage, as shown in Supplementary Fig. 9. Briefly, there are three types of nodes in the physical layer. The task manager monitors the whole process and assigns different tasks to the main workers and slaves. The main workers focus on one target variable and ensure that the tasks are executed in the right order. It also informs the task manager about the parallel tasks that are available. The slaves run parallel tasks assigned by the task manager. When a main worker finishes its job, it will turn into a slave node.

Supplementary Notes

Supplementary Note 1: Full comparison results on simulated datasets

Linear mixed-effects (LME) model

Supplementary Table 5. Model inference results for simulated data with 20 cases and 20 controls, noise variance $\sigma_\epsilon^2 = 3$ and samples taken every 3 months. Rows show the number of times each model is inferred as the best model using LME. 'Others' corresponds to all the other 27 possible AGPM models. The last two columns show the number of times the *diseaseAge* covariate has or has not been included in the final model

Generated \ Predicted	AGPM1	AGPM2	AGPM3	AGPM4	AGPM5	Others	*diseaseAge* included	*diseaseAge* not included
AGPM1	**6**	42	8	2	4	38	17	83
AGPM2	1	**57**	8	7	3	24	16	84
AGPM3	0	12	**51**	1	5	31	13	87
AGPM4	0	2	3	**19**	3	73	63	37
AGPM5	0	0	4	6	**18**	72	56	44

Supplementary Table 6. Model selection accuracy as a function of noise variance. Table shows the number of times the correct model is identified using LME.

Generated Datasets	noise = 1	noise = 3	noise = 5	noise = 8
AGPM1	10	6	16	13
AGPM2	64	57	57	54
AGPM3	62	51	48	52
AGPM4	16	19	19	20
AGPM5	15	18	21	11

Supplementary Table 7. Model selection accuracy as a function of sample size. Table shows the number of times the correct model is identified using LME.

Generated Datasets	10 cases and 10 controls	20 cases and 20 controls	30 cases and 30 controls	40 cases and 40 controls
AGPM1	30	6	7	1
AGPM2	53	57	56	55
AGPM3	38	51	63	64
AGPM4	14	19	12	9
AGPM5	15	18	15	12

Supplementary Table 8. Model selection accuracy as a function of sampling time points. Table shows the number of times the correct model is identified using LME.

Generated Datasets	2 months	3 months	4 months	6 months
AGPM1	8	6	10	0
AGPM2	50	57	57	60
AGPM3	42	51	62	53
AGPM4	16	19	16	26
AGPM5	17	18	13	16

Supplementary Table 9. Inclusion of *diseaseAge* in the final model for simulated data with 20 cases and 20 controls, noise variance $\sigma_\epsilon^2 = 3$ and samples taken every 3 months. Table shows the number of times the *diseaseAge* covariate is included in the inferred model using LME.

Generated Datasets	*diseaseAge* detected	*diseaseAge* not detected
AGPM1	17	83
AGPM2	16	84
AGPM3	13	87
AGPM4	63	37
AGPM5	56	44

Supplementary Table 10. Inclusion of *diseaseAge* in the final model as a function of noise variance. Table shows the number of times the *diseaseAge* covariate is included in the inferred model using LME.

Generated Datasets	noise = 1	noise = 3	noise = 5	noise = 8
AGPM1	11	17	18	13
AGPM2	17	16	18	12
AGPM3	8	13	19	12
AGPM4	55	63	66	61
AGPM5	62	56	60	63

Supplementary Table 11. Inclusion of *diseaseAge* in the final model as a function of sample size. Table shows the number of times the *diseaseAge* covariate is included in the inferred model using LME.

Generated Datasets	10 cases and 10 controls	20 cases and 20 controls	30 cases and 30 controls	40 cases and 40 controls
AGPM1	15	17	16	15
AGPM2	21	16	15	17
AGPM3	15	13	14	13
AGPM4	52	63	65	64
AGPM5	57	56	62	66

Supplementary Table 12. Inclusion of *diseaseAge* in the final model as a function of sampling time points. Table shows the number of times the *diseaseAge* covariate is included in the inferred model using LME.

Generated Datasets	2 months	3 months	4 months	6 months
AGPM1	19	17	12	19
AGPM2	17	16	12	17
AGPM3	22	13	10	14
AGPM4	63	63	64	61
AGPM5	59	56	52	46

Linear mixed-effects model (LME) with polynomial terms

Supplementary Table 13. Model inference results for simulated data with 20 cases and 20 controls, noise variance $\sigma_\epsilon^2 = 3$ and samples taken every 3 months. Rows show the number of times each model is inferred as the best model using LME with polynomial terms. 'Others' corresponds to all the other 27 possible AGPM models. The last two columns show the number of times the *diseaseAge* covariate has or has not been included in the final model

Generated \ Predicted	AGPM1	AGPM2	AGPM3	AGPM4	AGPM5	Others	*diseaseAge* included	*diseaseAge* not included
AGPM1	**10**	37	6	3	2	42	12	88
AGPM2	0	**50**	9	2	1	38	10	90
AGPM3	0	14	**41**	1	4	40	12	88
AGPM4	0	0	0	**25**	5	70	72	28
AGPM5	0	1	2	6	**19**	72	64	36

Supplementary Table 14. Model selection accuracy as a function of noise variance. Table shows the number of times the correct model is identified using LME with polynomial terms.

Generated Datasets	noise = 1	noise = 3	noise = 5	noise = 8
AGPM1	12	10	18	18
AGPM2	51	50	51	52
AGPM3	54	41	47	52
AGPM4	25	25	22	19
AGPM5	21	19	16	16

Supplementary Table 15. Model selection accuracy as a function of sample size. Table shows the number of times the correct model is identified using LME with polynomial terms.

Generated Datasets	10 cases and 10 controls	20 cases and 20 controls	30 cases and 30 controls	40 cases and 40 controls
AGPM1	35	10	6	3
AGPM2	42	50	50	46
AGPM3	34	41	51	58
AGPM4	18	25	17	15
AGPM5	17	19	14	15

Supplementary Table 16. Model selection accuracy as a function of sampling time points. Table shows the number of times the correct model is identified using LME with polynomial terms.

Generated Datasets	2 months	3 months	4 months	6 months
AGPM1	11	10	13	2
AGPM2	44	50	58	51
AGPM3	46	41	58	59
AGPM4	17	25	20	21
AGPM5	17	19	17	20

Supplementary Table 17. Inclusion of *diseaseAge* in the final model for simulated data with 20 cases and 20 controls, noise variance $\sigma_\epsilon^2 = 3$ and samples taken every 3 months. Table shows the number of times the *diseaseAge* covariate is included in the inferred model using LME with polynomial terms.

Generated Datasets	*diseaseAge* detected	*diseaseAge* not detected
AGPM1	12	88
AGPM2	10	90
AGPM3	12	88
AGPM4	72	28
AGPM5	64	36

Supplementary Table 18. Inclusion of *diseaseAge* in the final model as a function of noise variance. Table shows the number of times the *diseaseAge* covariate is included in the inferred model using LME with polynomial terms.

Generated Datasets	noise = 1	noise = 3	noise = 5	noise = 8
AGPM1	9	12	8	12
AGPM2	10	10	12	15
AGPM3	11	12	12	10
AGPM4	66	72	69	62
AGPM5	70	64	60	57

Supplementary Table 19. Inclusion of *diseaseAge* in the final model as a function of sample size. Table shows the number of times the *diseaseAge* covariate is included in the inferred model using LME with polynomial terms.

Generated Datasets	10 cases and 10 controls	20 cases and 20 controls	30 cases and 30 controls	40 cases and 40 controls
AGPM1	9	12	11	12
AGPM2	19	10	11	13
AGPM3	17	12	10	10
AGPM4	58	72	72	77
AGPM5	62	64	67	77

Supplementary Table 20. Inclusion of *diseaseAge* in the final model as a function of sampling time points. Table shows the number of times the *diseaseAge* covariate is included in the inferred model using LME with polynomial terms.

Generated Datasets	2 months	3 months	4 months	6 months
AGPM1	9	12	9	9
AGPM2	20	10	7	12
AGPM3	10	12	8	8
AGPM4	70	72	68	58
AGPM5	62	64	65	55

Gaussian process with automatic relevance determination (ARD) kernel

Supplementary Table 21. Model inference results for simulated data with 20 cases and 20 controls, noise variance $\sigma_\epsilon^2 = 3$ and samples taken every 3 months. Rows show the number of times each model is inferred as the best model using GP with ARD kernel. 'Others' corresponds to all the other 27 possible AGPM models. The last two columns show the number of times the *diseaseAge* covariate has or has not been included in the final model

Generated \ Predicted	AGPM1	AGPM2	AGPM3	AGPM4	AGPM5	Others	*diseaseAge* included	*diseaseAge* not included
AGPM1	**92**	0	0	0	0	8	0	100
AGPM2	0	**88**	2	0	0	10	2	98
AGPM3	0	60	**31**	1	1	7	9	91
AGPM4	0	18	0	**47**	4	31	71	29
AGPM5	0	19	11	15	**21**	34	54	46

Supplementary Table 22. Model selection accuracy as a function of noise variance. Table shows the number of times the correct model is identified using GP with ARD kernel.

Generated Datasets	noise = 1	noise = 3	noise = 5	noise = 8
AGPM1	93	92	90	91
AGPM2	90	88	81	72
AGPM3	30	31	35	47
AGPM4	62	47	47	30
AGPM5	16	21	18	20

Supplementary Table 23. Model selection accuracy as a function of sample size. Table shows the number of times the correct model is identified using GP with ARD kernel.

Generated Datasets	10 cases and 10 controls	20 cases and 20 controls	30 cases and 30 controls	40 cases and 40 controls
AGPM1	86	92	98	99
AGPM2	71	88	88	96
AGPM3	38	31	27	25
AGPM4	24	47	49	44
AGPM5	40	21	11	6

Supplementary Table 24. Model selection accuracy as a function of sampling time points. Table shows the number of times the correct model is identified using GP with ARD kernel.

Generated Datasets	2 months	3 months	4 months	6 months
AGPM1	94	92	86	88
AGPM2	91	88	75	75
AGPM3	29	31	35	41
AGPM4	53	47	42	28
AGPM5	12	21	25	21

Supplementary Table 25. Inclusion of *diseaseAge* in the final model for simulated data with 20 cases and 20 controls, noise variance $\sigma_\epsilon^2 = 3$ and samples taken every 3 months. Table shows the number of times the *diseaseAge* covariate is included in the inferred model using GP with ARD kernel.

Generated Datasets	*diseaseAge* detected	*diseaseAge* not detected
AGPM1	0	100
AGPM2	2	98
AGPM3	9	91
AGPM4	71	29
AGPM5	54	46

Supplementary Table 26. Inclusion of *diseaseAge* in the final model as a function of noise variance. Table shows the number of times the *diseaseAge* covariate is included in the inferred model using GP with ARD kernel.

Generated Datasets	noise = 1	noise = 3	noise = 5	noise = 8
AGPM1	0	0	0	0
AGPM2	3	2	8	12
AGPM3	1	9	5	5
AGPM4	70	71	73	63
AGPM5	71	54	64	55

Supplementary Table 27. Inclusion of *diseaseAge* in the final model as a function of sample size. Table shows the number of times the *diseaseAge* covariate is included in the inferred model using GP with ARD kernel.

Generated Datasets	10 cases and 10 controls	20 cases and 20 controls	30 cases and 30 controls	40 cases and 40 controls
AGPM1	1	0	0	0
AGPM2	12	2	7	1
AGPM3	5	9	3	6
AGPM4	71	71	81	78
AGPM5	75	54	67	75

Supplementary Table 28. Inclusion of *diseaseAge* in the final model as a function of sampling time points. Table shows the number of times the *diseaseAge* covariate is included in the inferred model using GP with ARD kernel.

Generated Datasets	2 months	3 months	4 months	6 months
AGPM1	0	0	2	3
AGPM2	0	2	11	13
AGPM3	1	9	8	10
AGPM4	73	71	67	66
AGPM5	50	54	58	53

Supplementary References

[1] Andrzej Galecki. *Linear Mixed-Effects Models Using R*. Springer Publishing Company, Incorporated, 2015.

[2] Radford M. Neal. *Bayesian Learning for Neural Networks*. Springer-Verlag, Berlin, Heidelberg, 1996.

[3] A. Gelman, J.B. Carlin, H.S. Stern, D.B. Dunson, A. Vehtari, and D.B. Rubin. *Bayesian Data Analysis, Third Edition*. Chapman & Hall/CRC Texts in Statistical Science. Taylor & Francis, 2013.
