# Supplementary material for: An additive Gaussian process regression model for interpretable non-parametric analysis of longitudinal data
Source: Nat Commun. 2019 Apr 17;10:1798. doi: 10.1038/s41467-019-09785-8 (PMC6470127; doi:10.1038/s41467-019-09785-8)
Supplement: Supplementary file 3 — Description of Additional Supplementary Files [file 41467_2019_9785_MOESM3_ESM.pdf]

Description of Additional Supplementary Files

File Name: Supplementary Data 1
Description: Model selection results of LME, LME-P, and GP-ARD over all simulated datasets.

File Name: Supplementary Data 2
Description: Full metagenomics dataset results including the selected models and explained variances of the components for all 394 pathways.

File Name: Supplementary Data 3
Description: Full proteomics dataset results including the selected models and explained variances of each component for all 1538 proteins.
